# Analysis of skeletal characteristics of flat feet using three-dimensional foot scanner and digital footprint

**DOI:** 10.1186/s12938-022-01021-7

**Published:** 2022-08-09

**Authors:** Tomoko Yamashita, Kazuhiko Yamashita, Mitsuru Sato, Masashi Kawasumi, Shingo Ata

**Affiliations:** 1 Graduate School of Engineering, Osaka City University, 3-3-138 Sugimoto, Sumiyoshi, Osaka, 558-8585 Japan; 2 Department of Clinical Engineering, Faculty of Human Care at Makuhari, Tohto University, Chiba, Japan; 3 School of Nursing and Rehabilitation Sciences, Showa University, Kanagawa, Japan; 4 School of Science and Technology for Future Life, Tokyo Denki University, Tokyo, Japan

**Keywords:** 3D foot scanning, Digital footprint, Flat feet, Navicular height, Axis of bone distance

## Abstract

**Background:**

Flat feet increase the risk of knee osteoarthritis and contribute to frailty, which may lead to worse life prognoses. The influence of the foot skeletal structure on flat feet is not yet entirely understood. Footprints are often used to evaluate feet. However, footprint-based measurements do not reflect the underlying structures of feet and are easily confounded by soft tissue. Three-dimensional evaluation of the foot shape can reveal the characteristics of flat feet. Therefore, foot shape evaluations have garnered increasing research interest. This study aimed to determine the correlation between the three-dimensional (3D) features of the foot and the measurement results of footprint and to predict the evaluation results of flat feet from the footprint based on the 3D features. Finally, the three-dimensional characteristics of flat feet, which cannot be revealed by footprint, were determined.

**Methods:**

A total of 403 individuals (40–89 years) participated in this study. The proposed system was developed to identify seven skeletal features that were expected to be associated with flat feet. The loads on the soles of the feet were measured in a static standing position and with a digital footprint device. Specifically, two footprint indices were calculated: the Chippaux–Smirak index (CSI) and the Staheli index (SI). In the analysis, comparisons between male and female measurement variables were performed using the Student’s *t* test. The relationships between the 3D foot features and footprint index parameters were determined by employing the Pearson correlation coefficient. Multiple linear regression was utilized to identify 3D foot features that were strongly associated with the CSI and SI. Foot features identified as significant in the multivariate regression analysis were compared based on a one-way analysis of variance (ANOVA) with Tukey’s post hoc test.

**Results:**

The CSI and SI were highly correlated with the instep height (IH) and navicular height (NH) of the 3D foot scanning system and were also derived from multiple regression analysis. In addition to the NH and IH, the indicators of the forefoot, transverse arch width, and transverse arch height were considered. In the flat foot group with CSI values above 62.7%, NH was 13.5% (*p* < 0.001) for males and 14.9% (*p* = 0.01) for females, and the axis of the bone distance was 5.3% (*p* = 0.05) for males and 4.9% (*p* = 0.10) for females. In particular, for CSI values above 62.7% and NH values below 13%, the axis of the bone distance was large and the foot skeleton was deformed.

**Conclusions:**

Decreased navicular bone height could be evaluated with the 3D foot scanning system even when flat feet were not detected from the footprint. The results indicate that the use of quantitative indices for 3D foot measurements is important when evaluating the flattening of the foot.

*Trial registration number* UMIN000037694.

Name of the registry: University Hospital Medical Information Network Registry.

Date of registration: August 15, 2019.

## Introduction

Flat feet increase the risk of knee osteoarthritis and contribute to frailty [1, 2], which may lead to worse life prognoses. Flatfoot is related to a lack of foot arch support and insufficient flexibility of the plantar ligaments and tendons [3, 4] and the collapse in the medial arch of the foot [5]. It reduces the ability to absorb the impact on the foot while walking or running and can increase the risk of foot injury and lead to plantar fasciitis, metatarsal pain, knee pain, lower back pain, hindfoot deformity such as osteoarthritis of the subtalar and Chopart joints because of the high impact forces [6–8]. Therefore, there is increasing interest in foot shape evaluations for flat feet [9, 10].

For diagnosing flatfoot, it is important to consider the following factors: the degree of severity of subjective symptoms; physical findings obtained during the clinical examination; analysis of the obtained footprint; and diagnostic imaging studies, which may include weightbearing radiographs, bone scans, computed tomography, and magnetic resonance imaging [3]. To determine the degree of deformity, a set of radiological parameters is used to measure the specific angles obtained by standard dorsoplantar and lateral radiographs of the weightbearing feet. The procedure of determining these angles is often difficult and expeditious, and it depends on a quality of X-ray and skill of the observer [11].

A typical parameter to evaluate flatfoot is navicular bone height [12]. In navicular bone height evaluation, there have been issues related to X-ray skills and hesitation to use X-ray evaluation due to ethical constraints in epidemiological studies targetting healthy adults and children. To assess the arch height of flat feet, researchers measure the height of the navicular bone manually [13, 14]. Thus, it is difficult to identify the navicular bone height.

Footprint analysis is a simple, cost-effective, and readily available method and has been recommended as a screening tool for flatfoot [6, 15]. Only three evaluations of flatfoot have any published data to support validity and reliability of the measuement: the Chippaux–Smirak index (CSI), Staheli index (SI) and the FPI-6 [16]. However, each of these measures were deemed to have limitations [16].

Previous studies have also evaluated the flatness of feet using the footprint-based CSI or SI [17, 18]. Furthermore, diagnostic accuracy has been verified by comparing the clinical diagnoses of flat feet to those based on CSI and SI for individuals aged 40 and above [19]. However, inconsistent results were obtained in measurements using calipers and ink mats [20, 21]. Furthermore, there are concerns that two-dimensional indices are limited in their ability to assess a three-dimensional (3D) construct [21]. Thus, footprint-based measurements do not reflect the underlying structure of the foot and are easily confounded by soft tissue.

To identify foot problems, it is necessary to estimate the three-dimensional foot skeletal characteristics and to obtain precise and quantitative measurements. The 3D dimensions of feet and their surface shapes have been captured in several previous studies [22–25]. Consequently, many scholars have measured the outer dimensions of the foot [26]. However, it is important to note that previous researchers have not estimated the indices of footprint for flatfoot from the 3D foot structure.

This study proposes a method for evaluating the shape of the foot without the use of special expensive equipment. In particular, a convenient smartphone-based foot scanner is presented. This study aims to determine the correlation between the 3D features of the foot and the measurement results of the footprint and to predict the evaluation results of flat feet using footprint based on the 3D features. Finally, the 3D characteristics of flat feet, which cannot be revealed by footprint, are determined.

## Results

### Differences based on gender and age-related changes

The measured results of the 3D foot-scanning system revealed differences based on gender for all indices except for the FFH angle (Table 1). The IH and NH, which are related to the skeletal structure of the midfoot region, presented larger values in males than in females. On the other hand, the TAW, which is related to the forefoot, was larger in the female participants. In addition, the GFH angle was 10% larger and the ABD was 16% higher in females than in males.

**Table 1:** Results of 3D foot-scan and footprint measurements

IH		NH	TAW	TAH	FFH angle	GFH angle	ABD
(a) Results for 3D foot feature index values							
Male	28.5 (2.3)	19.5 (2.9)	41.5 (2.0)	16.4 (1.5)	31.5 (4.9)	34.9 (5.5)	3.2 (2.4)
Female	27.3 (2.0)	17.7 (2.9)	42.5 (2.5)	16.0 (1.5)	31.1 (5.4)	38.2 (6.5)	3.7 (2.4)
* P* value 95% CI	0.01 0.61 to 5.42	0.01 1.17 to 2.36	0.01 −1.47 to −0.52	0.01 0.14 to 0.75	0.46 −0.65 to 1.43	0.01 −4.58 to −2.10	< 0.001 −2.38 to −1.36

Forefoot		Midfoot	Heel	CSI	SI
(b) Results for footprint index values					
Male	44.8 (2.6)	18.6 (5.6)	24.0 (2.1)	41.8 (11.6)	0.78 (0.24)
Female	46.8 (3.0)	20.8 (5.9)	25.3 (2.2)	44.8 (11.7)	0.83 (0.25)
* P* value 95% CI	0.01 −2.51 to −1.40	0.01 −3.34 to −1.03	0.01 −1.77 to −0.90	0.01 −5.30 to −0.58	0.07 −0.10 to −0.004

All data are shown as means ± standard deviations (SD)

*IH* Instep height; *NH* Navicular height; *TAW* Transverse arch width; *TAH* Transverse arch height; *FFH* Fifth toe–fifth metatarsal head–heel; *GFH* Great toe–first metatarsal head–heel; *ABD* axis of the bone distance; *CSI* Chippaux–Smirak index; SI: Staheli index

Differences based on gender were confirmed for all parameters except for the SI. The forefoot corresponding to the TAW was 2% larger, and the midfoot was 12% larger in females than in males (Table 1b). Neither the 3D foot-scanning system nor the footprint yielded correlations between age and any of the other tested variables (see Table 2), except for the TAW, in males. By contrast, various parameters were weakly correlated in females. The forefoot demonstrated a strong correlation with the TAW (0.60 for males, 0.56 for females, *p* < 0.01) and a weak correlation with the GFH angle, which is related to the hallux valgus (0.19 for males, *p* < 0.05; 0.21 for females, *p* < 0.01). The midfoot, CSI, and SI exhibited high correlations with the IH and NH. Moreover, a correlation between the GFH angle and CSI for the male participants (0.30, *p* < 0.01) was revealed. No correlation was found between the CSI and GFH angle in females.

**Table 2:** Correlations between 3D foot features and footprint indices

Male	Age	BMI	IH	NH	TAW	TAH	FFH	GFH	ABD
Age	1	−0.18	0.08	0.03	0.16^a^	0.09	0.05	0.10	0.14
BMI	−0.18^a^	1	0.21^a^	0.13	0.1	0.21^a^	−0.03	−0.01	−0.1
CSI	0.03	0.10	−0.53^b^	−0.56^b^	0.14	−0.13	0.04	0.30^b^	0.18^a^
SI	0.02	0.09	−0.52^b^	−0.58^b^	0.16	−0.2^b^	0.11	0.27^b^	0.15
Forefoot	0.15	0.01	0.05	0.02	0.60^b^	0.18^a^	0.14	0.19^a^	0.01
Midfoot	0.08	0.09	−0.51^b^	−0.54^b^	0.27^b^	−0.10	0.07	0.35^b^	0.17^a^
Heel	0.19^a^	−0.05	0.14	0.21^a^	0.32^b^	0.2^a^	−0.1	0.16^a^	0.07

Female	Age	BMI	IH	NH	TAW	TAH	FFH	GFH	ABD
Age	1	−0.09	−0.15	−0.11	0.23	−0.18	0.03	0.25	0.19
BMI	−0.09	1	0.09	0.01	0.17^d^	0.46^d^	0.15^c^	0.00	−0.07
CSI	0.14^c^	0.27^d^	−0.41^d^	−0.41^d^	0.15^c^	−0.06	−0.09	0.12	0.13^c^
SI	0.21^d^	0.24^d^	−0.41^d^	−0.39^d^	0.22^c^	−0.10	−0.02	0.15^c^	0.08
Forefoot	0.21^d^	0.00	0.03	0.06	0.56^d^	0.03	0.18^d^	0.21^d^	0.00
Midfoot	0.21^d^	0.25^d^	−0.38^d^	−0.37^d^	0.29^d^	−0.05	0.00	0.16^d^	0.11
Heel	−0.02	0.07	0.17^d^	0.13^c^	0.17^d^	0.21^d^	0.09	0.02	0.07

Pearson coefficient (^a^*p* < 0.05, ^b^*p* < 0.01). Listed results are for the male/female patricipants

Pearson coefficient (^c^*p* < 0.05, ^d^*p* < 0.01). Listed results are for female participants

### Three-dimensional foot measurement parameters that predict CSI and SI based on gender

Table 3 presents the regression results for the relationships between four independent foot features—the NH, IH, BMI, and TAW—and the CSI for males and females. Table 4 shows the regression results for the relationships between these four features and the SI for both gender groups. The NH, TAW, IH, and BMI were similar for both gender groups. Age was also a feature of interest for the female group. The adjusted *r*^*2*^ values denoting the strength of the relationship were measured to be 0.43 and 0.32 for the male and female groups, respectively.

**Table 3:** Multiple regression analysis results for the CSI

Variable	B	SE B	*β*	*p* value	*r*^*2*^	Adjusted *r*^*2*^
NH	−0.014/−0.008	0.004/0.003	−0.355/−0.203	< 0.001/0.01	0.43/0.32	0.41/0.31
IH	−0.018/−0.019	0.005/0.004	−0.344/−0.333	< 0.001/ < 0.001	*F* value	*P* value
BMI	0.007/0.009	0.002/0.002	0.206/0.272	< 0.001/ < 0.001	55.60/28.50	< 0.001/ < 0.001
TAW	0.010/0.008	0.004/0.002	0.179/0.176	0.01/ < 0.001	–	–

Pearson coefficient (^a^*p* < 0.05, ^b^*p* < 0.01). Listed results are for the male patricipants

**Table 4:** Multiple regression analysis results for the SI

Variable	B	SE B	*β*	*p* value	*r*^*2*^	Adjusted *r*^*2*^
NH	−0.035/-0.015	0.008/0.006	−0.425/−0.168	< 0.001/0.02	0.44/0.34	0.43/0.32
TAW	0.023/0.023	0.008/0.006	0.191/0.227	0.003/ < 0.001	F value	*p* value
IH	−0.03/−0.043	0.01/0.009	−0.281/−0.344	0.003/ < 0.001	28.48/25.03	< 0.001/ < 0.001
BMI	0.013/0.016	0.005/0.004	0.182/0.239	0.005/ < 0.001	–	–
Age (female)	0.002	0.001	0.109	0.05		

Pearson coefficient (^a^*p* < 0.05, ^b^*p* < 0.01). Listed results are for the female patricipants

### One-way ANOVA results and 3D midfoot parameter distribution related to CSI

**Fig. 1:**
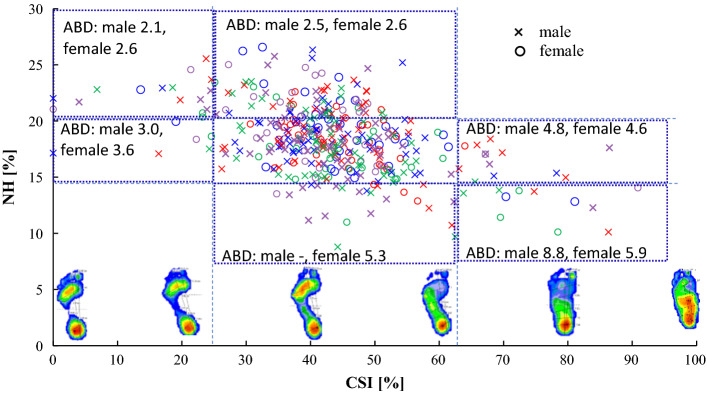
Scatter plot of NH versus CSI. The plots represent the ABD quartiles. Blue: first quartile. Red: second quartile. Green: third quartile. Purple: fourth quartile

Previous studies on flat feet have focused on the CSI and NH [27, 28]. In this study, the NH, IH, and ABD were regarded as the factors necessary to investigate the relationship between the height of the MLA and CSI. Table 5 summarizes the results of a one-way ANOVA for the CSI in relation to the NH, IH, and ABD, and Fig. 1 shows a scatter plot of the CSI and NH. For the CSI, values ≥ 62.7 qualify as flat feet, whereas values ≤ 25 qualify as high arches [27, 28]. Therefore, in this study, we conducted classifications based on this criterion and performed one-way ANOVA analyses of the NH, IH, and ABD according to gender.

**Table 5:** One-way analysis of variance of 3D foot measurements results for the CSI

CSI		Number of participants		NH				IH	
		Male	Female	Male	*p* value	Female	*p* value	Male	*p* value
> 62.7	Flat feet	7	17	14.5 (1.6)	< 0.001	14.9 (2.7)	0.01	25.5 (2.2)	< 0.001
> 25	Normal	132	225	19.5 (2.7)	0.003	17.8 (2.8)	0.02	28.4 (2.0)	< 0.001
≤ 25	Higher arch	12	10	22.2 (1.9)	< 0.001	20.1 (2.4)	< 0.001	30.7 (2.5)	< 0.001

CSI		IH		ABD			
		Female	*p* value	Male	*p* value	Female	*p* value
> 62.7	Flat feet	25.9 (2.0)	< 0.001	5.3 (4.3)	0.05	4.9 (3.1)	0.10
> 25	Normal	27.3 (2.0)	< 0.03	3.2 (2.3)	0.34	3.6 (2.3)	0.78
≤ 25	Higher arch	29.1 (1.5)	< 0.001	2.2 (1.2)	0.01	3.1 (2.0)	0.15

*p* value: upper: between normal and flat feet, middle: between normal and higher arches, lower: between flat feet and higher arches. Results are given as mean (SD)

The NH classification on the vertical axis of Fig. 1 correspond to the values for females in Table 5. In the results, the NH and IH differ significantly between flat, normal, and high-arched feet, as determined using the CSI. In males, the ABD values show that flat feet indicate a large displacement of the foot skeletal structure.

## Discussion

In this study, a system that can easily measure the 3D foot structure was developed and the footprint measurement results were employed to analyze the characteristics of flat feet based on gender and age. Furthermore, it was determined that the skeleton of the foot is related to the footprint index.

It was found that the ABD, GFH angle, and TAW were larger, whereas the NH and TAH were smaller in females than in males. The CSI and SI were highly correlated with the IH and NH features of the 3D foot-scanning system and were also derived using multiple regression analyses. Furthermore, navicular bone and foot height were found to be related to the midfoot skeletal structure. Consequently, the CSI and SI were confirmed to be correlated with the IH and NH. Previous studies have reported correlations between the NH and flat feet [11, 29].

The mechanism responsible for flat and pronated feet is related to changes in the position of the skeletal structure, such as that of the navicular bone. Given that there exists a moderate correlation between the footprint index and 3D foot surface structure, the skeletal features of the foot cannot be evaluated adequately by utilizing footprint indices alone.

Flat feet are typically combined with forefoot abduction, rear-foot eversion, collapse of the MLA, foot abduction at the talonavicular joint, and subtalar joint eversion [30–32]. Therefore, the midfoot is flattened due to the lowering of the navicular bone, which was the primary factor that affected the prediction accuracy of flat feet in this study. When the forefoot was pronated, the height of the navicular bone decreased; however, given that the bottom of the foot did not touch the ground, it was not detected as a flatfoot based on the footprint. Therefore, even if the CSI or SI indicated that the foot was not flat, the navicular bone was lowered. Moreover, there was an increased likelihood that the foot was pronated, which could be evaluated using the 3D foot-scanning system. Fig. 1 shows that as the CSI increases, the ABD increases as well; the ABD can only be revealed by 3D analysis of the foot.

The results obtained by the multiple regression analysis of the CSI and SI showed that in addition to the NH and IH, the TAW and TAH act as forefoot indicators. Furthermore, the results suggest that the transverse and medial longitudinal arches are important for predicting both the CSI, focused on the forefoot, and the SI, focused on the hindfoot.

The forefoot and midfoot, as identified from the footprint, were weakly correlated with the NH, IH, and TAW in females. Table 1 indicates that in females, forefoot flattening occurs due to the lowered position of the navicular bone and the effect of the hallux valgus, because the ABD, which reflects the GFH angle associated with the hallux valgus and the skeletal deviation of the foot, was 15% higher in females than in males. Skeletal deformities that occur with age also influenced the walking pattern and gait. To date, no reports have been published on the distortion of the skeletal structure of the foot. To predict and prevent these foot problems, the 3D structure of the foot should be measured and evaluated in addition to the footprint.

The prevalence of flexible flat feet was estimated to be 13.6% based on the navicular drop test results [12]. The prevalence of flat feet based on the footprints was 19.0% and increased with age [13]. In this study, 4.6% of males and 6.7% of females were identified as having flat feet based on the CSI, and 7.9% of males and 4.0% of females were identified as having high arches. These prevalence values are lower than those measured in previous studies.

From the scatter plot shown in Fig. 1, a group with NH values < 13% was identified and classified as having normal CSI values between 25 and 62.7% Based on the ABD value (5.29%), the skeleton was tilted inward, leading to moderate pronation of the foot. Similarly, in the group with CSI values above 62.7% and NH values below 13%, the ABD was 8.78% for males and 5.91% for females. It is presumed that foot flattening occurred due to the large skeletal deformation of the foot. Further classification of the NH suggested that as the NH increased, the ABD decreased, indicating less skeletal misalignment. This characteristic has not been revealed in previous studies.

The above discussion suggests that foot characteristics can be evaluated using the foot structural features from the 3D foot-surface-structure scanning system.

## Conclusions

In this study, we compared the indices of the 3D foot-scanning system with digital footprints and investigated their correlations with foot skeletal features. The results indicated that the indices of the 3D foot-scanning system contribute moderately to the CSI and SI flatfoot indices associated with digital footprints. Furthermore, based on the index values of the 3D foot-scanning system, it was inferred that pronation of the midfoot results in flat feet. Thus, the importance of using quantitative indices for 3D foot measurements for foot flattening evaluation was proven.

The superior performance of the 3D foot-scanning system is attributable to the fact that the surface shape of the foot can be evaluated using a simple, quantitative method that does not require special equipment. By evaluating the shape of the foot, which has many diverse characteristics, we hope to enable preventative treatments for flat feet that help extend the healthy life span of the foot.

## Methods

### Participants

The study design was approved by the Ethical Review Board at Tohto University (Authorization number: R0306). The study was conducted in accordance with the tenets of the Declaration of Helsinki. All participants signed informed consent forms prior to participation. The participants consisted of 403 individuals in the age range of 40–89 years. They were recruited via advertisements to participate in this study. The inclusion criterion required the participants to be capable of walking without assistance. The exclusion criteria were musculoskeletal disorders of the lower extremities and major lower limb trauma. The participant characteristics are listed in Table 6.

**Table 6:** Participant characteristics.

	Number of participants	Age	Height (cm)	Weight (kg)	Body mass index (BMI)
Male	151	65.6 (11.0)	166.9 (5.7)	67.1 (10.4)	24.1 (3.3)
Female	252	62.6 (12.4)	154.5 (6.0)	55.0 (9.3)	23.0 (3.7)
*P* value 95% confidence interval (CI)	–	0.01 0.61–5.42	–	–	< 0.001 0.33–1.73

(a) Basic characteristics and number of participants

## Measurement methods

### Analysis of foot-surface structure

In this study, we developed a 3D foot measurement system using a smartphone (iPhone 6, Apple Inc., Cupertino, CA, USA) and employed this smartphone-based system to measure the foot surface structure. The developed 3D foot scanner analyzed the foot features of the participants through videos obtained using a smart device camera [24, 25]. The system showed a spatial resolution of 1.7 mm with high reproducibility of results [24].

**Fig. 2:**
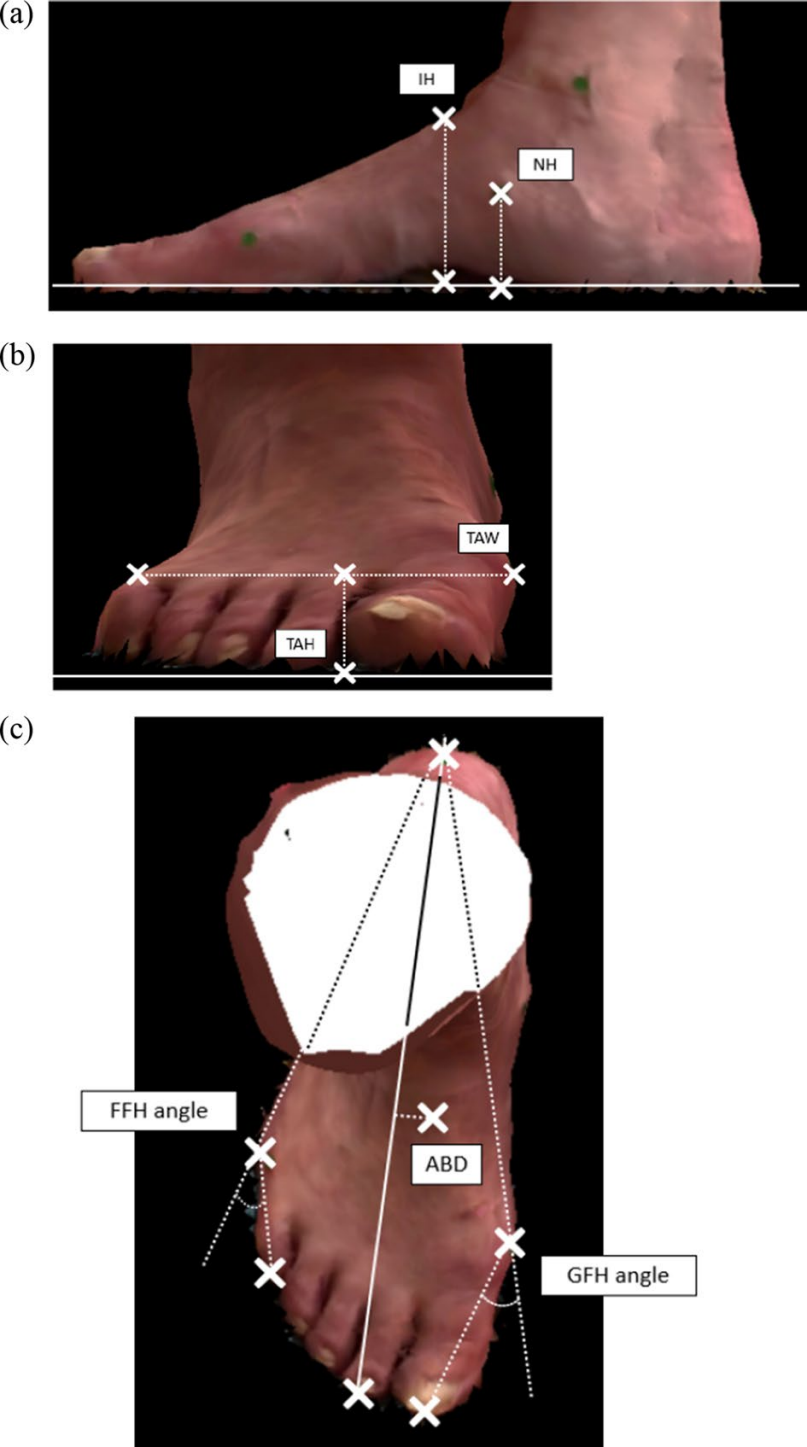
Seven foot features of the three-dimensional (3D) foot-scanning system. **a** Instep height (IH) and navicular height (NH). **b** Transverse arch height (TAH) and transverse arch width (TAW). **c** Great toe–first metatarsal head–heel (GFH) angle, fifth toe–fifth metatarsal head–heel (FFH) angle, and distance from the center line between the heel and the second-toe tip when the coordinate point of the talus head is projected onto the floor (ABD)

The foot feature points were identified by a physical therapist. In this study, the 3D foot-surface model was based on the measured results for the left foot. Figure 2 shows the seven foot features assessed in this study. Previously, we measured the feet of middle-aged and older adults using this measurement system, reported the changes in the feet due to aging and the characteristics of the feet related to the hallux valgus, and discussed the effectiveness of this measurement system [25]. In this study, the same index was used for evaluation.

Figure 2(a) shows the instep height (IH) and navicular height (NH); Fig. 2(b) illustrates the transverse arch width (TAW) and height (TAH); and Fig. 2(c) depicts the great toe–first metatarsal head–heel (GFH) angle, fifth toe–fifth metatarsal head–heel (FFH) angle, and axis of the bone distance (ABD). The ABD is the centerline distance between the heel and tip of the second toe when the talus-head coordinate is projected onto the floor. Medial longitudinal arches can be assessed using the IH and NH. In addition, the TAW and TAH can be considered as indices for measuring the transverse arch. The GFH and FFH angles reflect the hallux valgus and digitus minimus varus indicators, respectively, and the ABD indicates the foot pronation [25].

### Measurement of digital footprint index

**Fig. 3:**
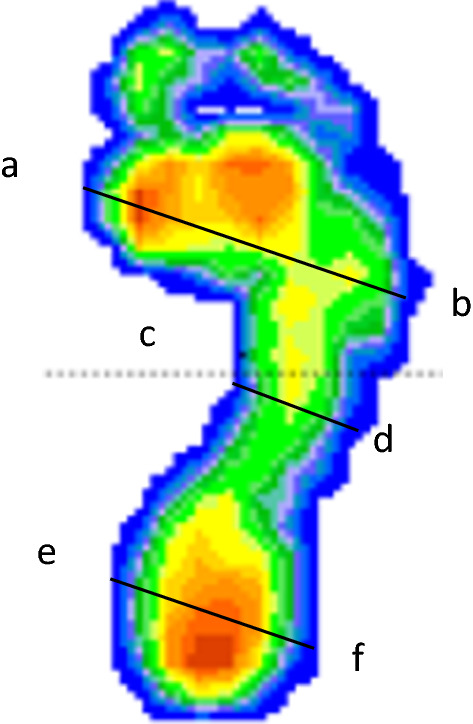
Graphical representation of the Chippaux–Smirak and Staheli indices (CSI and SI, respectively). **a**: interior of the first metatarsal head, b: exterior of the fifth metatarsal. Line (**c**–**d**): line of minimal length across the arch parallel to line (**a**–**b)**. Line **e**–**f**: parallel line used to represent the heel width

The loads on the soles of the feet were measured using a digital footprint device with the participants in a static standing position. Fig. 3 shows the parts used for analysis: (i) forefoot: region between the interior of the first metatarsal head and exterior of the fifth metatarsal (line a–b), (ii) midfoot: identified by a parallel line representing the minimal width of the foot in the area of the arch (line c–d), and (iii) heel: identified by a parallel line representing the heel width (line e–f).

Specifically, two footprint indices were calculated: the CSI {midfoot [line(c–d)] × 100/forefoot [line(a–b)]} and SI {midfoot [line(c–d)]/heel [line(e–f)]}. The CSI is the ratio of the minimum width of the midfoot arch region to the maximum width of the forefoot region [13, 33, 34], and the SI is the ratio of the minimum width of the midfoot arch region to the maximum width of the rear-foot region [12, 13, 18, 19, 26].

The distances based on the 3D foot and footprint indices are affected by the foot length. Therefore, the values of the parameters used in the study were normalized with respect to the distance between the heel and tip of the second toe as an indicator of the foot length. The distances were also multiplied times 100. In addition, the effect of the patient physique was evaluated by measuring the height and weight of each participant to calculate their body mass index (BMI).

### Statistical analysis

In this study, statistical software was used to analyze the measured data. The quantitative variables were expressed as mean values (± standard deviation (SD)). In the analysis, comparisons between male and female measurement variables were conducted using the Student’s *t* test. The relationships between the 3D foot features and footprint index parameters were explored by employing the Pearson correlation coefficient. Multiple linear regression was utilized to identify the 3D foot features that were strongly associated with the CSI and SI. Foot features identified as significant in the multivariate regression analysis were compared by performing a one-way analysis of variance (ANOVA) with Tukey’s post hoc test. The significance was set to *p* ≤ 0.05 for all tests.

## Data Availability

The data sets used or analyzed during the current study are available from the corresponding author on reasonable request.
